# Oral d-ribose causes depressive-like behavior by altering glycerophospholipid metabolism via the gut-brain axis

**DOI:** 10.1038/s42003-023-05759-1

**Published:** 2024-01-09

**Authors:** Ke Xu, Yi Ren, Shuang Zhao, Jinzhou Feng, Qingyuan Wu, Xue Gong, Jianjun Chen, Peng Xie

**Affiliations:** 1 Department of Neurology, The First Affiliated Hospital of Chongqing Medical University, 400016 Chongqing, China; 2 National Health Commission Key Laboratory of Diagnosis and Treatment on Brain Functional Diseases, The First Affiliated Hospital of Chongqing Medical University, 400016 Chongqing, China; 3 Department of Infectious Diseases, Key Laboratory of Molecular Biology for Infectious Diseases, Ministry of Education, Institute for Viral Hepatitis, The Second Affiliated Hospital of Chongqing Medical University, 400010 Chongqing, China; 4 Lab of Stem Cell and Tissue Engineering, Department of Histology and Embryology, 400016 Chongqing, China; 5 Department of Neurology, Chongqing University Three Gorges Hospital, 404031 Chongqing, China; 6 Institute of Life Sciences, Chongqing Medical University, 400016 Chongqing, China

**Keywords:** Depression, Microbiology

## Abstract

Our previous work has shown that d-ribose (RIB)-induced depressive-like behaviors in mice. However, the relationship between variations in RIB levels and depression as well as potential RIB participation in depressive disorder is yet unknown. Here, a reanalysis of metabonomics data from depressed patients and depression model rats is performed to clarify whether the increased RIB level is positively correlated with the severity of depression. Moreover, we characterize intestinal epithelial barrier damage, gut microbial composition and function, and microbiota-gut-brain metabolic signatures in RIB-fed mice using colonic histomorphology, 16 S rRNA gene sequencing, and untargeted metabolomics analysis. The results show that RIB caused intestinal epithelial barrier impairment and microbiota-gut-brain axis dysbiosis. These microbial and metabolic modules are consistently enriched in peripheral (fecal, colon wall, and serum) and central (hippocampus) glycerophospholipid metabolism. In addition, three differential genera (Lachnospiraceae_UCG-006, Turicibacter, and Akkermansia) and two types of glycerophospholipids (phosphatidylcholine and phosphatidylethanolamine) have greater contributions to the overall correlations between differential genera and glycerophospholipids. These findings suggest that the disturbances of gut microbiota by RIB may contribute to the onset of depressive-like behaviors via regulating glycerophospholipid metabolism, and providing new insight for understanding the function of microbiota-gut-brain axis in depression.

## Abstract (web summary)

A combined colonic histomorphology, 16 S rRNA gene sequencing, and untargeted metabolomics analysis on D-ribose-fed mice suggest that oral D-ribose caused intestinal epithelial barrier impairment and microbiota-gut-brain axis dysbiosis.

## Introduction

D-ribose (RIB) is a naturally occurring monosaccharide that is found in riboflavin-containing foods such as wheat bran, eggs, and meat. Meanwhile, because RIB can bypass part of the pentose pathway to produce d-ribose-5-phosphate for the production of energy, it has been utilized as a daily nutritional or energy supplement^1^, notably for patients with chronic fatigue syndrome and coronary artery disease^2,3^. RIB is also a crucial component of several important biomolecules including adenosine and adenosine triphosphate, which are involved in a variety of metabolic activities^4^. However, as described by the European Food Safety Authority, the toxicological effects of RIB should not be ignored^5^. Several studies have reported that RIB can be involved in the onset of encephalopathy^6,7^.

Depression is one of the most prevalent serious mental disorders, characterized by a lack of interest, pessimism, appetite loss, and even suicidal behavior^8,9^. A new epidemiology study estimates that 7% of people experience depression annually, with a lifetime prevalence of over 15%^10^. Finding relevant risk variables is crucial for depression prevention and screening. Given that RIB has not been widely reported in depressive disorder and that a high-sugar diet may be an environmental risk factor for depression^11,12^, we recently gave normal mice prolonged RIB supplementation and found that these mice exhibited depressive-like behaviors and histological alterations, including obviously condensed and deeply-stained pyramidal cells in the hippocampus^13^. This finding implies that RIB has a significant impact on the development of depression. The key scientific concerns that we will research, however, are whether the variation in RIB level was associated with depression and the underlying biological mechanism of RIB implicated in depressive illness.

Of note, some other monosaccharides, such as fructose and glucose, have been linked to changes in the gut microbiota, leading to microbial metabolite disorder in rodents^14,15^. A high intake of sugar can cause enteric dysbacteriosis. The latter increases the permeability of the intestinal mucosa, and results in abnormalities in intestinal immunity and glucolipid metabolism^15^. Moreover, hyperglycemia would increase the permeability of the intestinal barrier, giving microbes a better chance to enter the body and causing the proliferation of pathogenic bacteria^16^. Nearly every aspect of host physiology may be influenced by gut microbiota, from controlling gut metabolism to influencing mood and behavior via the “microbiota-gut-brain” (MGB) axis^17,18^. Our groups previously found that depression was linked to altered gut microbiomes^19^, and germ-free mice exhibit depressive-like behaviors after receiving gut microbiota from depressed patients^19,20^. Furthermore, a recent study demonstrates that exogenous RIB can affect gut microbial architecture^21^. As a result, we proposed that changes in microbiota might account for the connection between RIB intake and depression.

At first, we reanalyzed the metabonomics data from our earlier studies to clarify the change of RIB in the urine of depressed patients and in the hippocampus of depression model rats. Then, in the current study, to further investigate the possible mechanism of RIB-induced depression, eight weeks of RIB-fed mice were constructed. The intestinal barrier impairment was evaluated using hematoxylin and eosin, immunohistochemistry, and electron microscopy. The distinct gut microbiota was initially identified by 16S rRNA gene sequencing analysis. Moreover, by systematic analysis of relevant biological samples, including peripheral (fecal, colon wall, and serum) and central (hippocampus) specimens from the RIB-fed mice and control mice, comparative untargeted metabolomics was used to capture the functions of the altered gut microbiome. Finally, by integrating these multi-omics data, we sought to understand how the gut microbiota contributed to the development of depressive-like behaviors and to pinpoint a putative way between the gut and the brain in RIB-fed mice.

## Results

### RIB was significantly increased in depressed patients and depression model rats

**Fig. 1:**
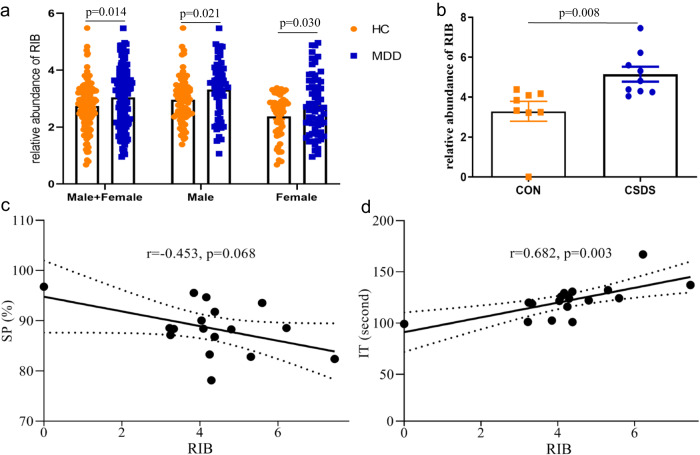
Increased levels of d-ribose (RIB) in both depressed patients and depression model rats. **a** The relative abundance of ribose was significantly increased in patients with major depressive disorder (MDD; *n* = 126) compared to healthy controls (HC; *n* = 125); the relative abundance of RIB in male (*n* = 63) and female (*n* = 63) MDD patients was significantly different from of that in their respective HC (male, *n* = 77; female, *n* = 48). **b** The relative abundance of RIB was significantly increased in chronic social defeat stress (CSDS) model rats (*n* = 9) compared to control (CON) rats (*n* = 8). **c** There was a negative relationship between the percentage of sucrose preference (SP) and the relative abundance of RIB. **d** There was a significant positive correlation between immobility time (IT) and the relative abundance of RIB. Data are the means ± standard error of mean. The group-level comparisons were conducted using Student’s *t*-test to assess the changes of RIB in different groups, and Spearman’s correlation analysis was used to assess the relationships between RIB level and depressive-like behaviors.

Compared to healthy controls (HC), the relative abundance of RIB was significantly increased in the urine of depressed patients (*p* = 0.014; Fig. 1a). Meanwhile, we found that the levels of RIB in males (*p* = 0.021) and females (*p* = 0.030) with depression were both significantly different from that in their respective HC. Depression model rats have greater RIB levels in their hippocampus than control (CON) rats (*p* = 0.008; Fig. 1b). These group-level comparisons were conducted using Student’s *t*-test. The results of Spearman’s correlation analysis showed that although there was no significant correlation between sucrose preference and RIB levels (*r* = −0.453, *p* = 0.068; Fig. 1c) in the depression model rats, the immobility time was significantly positively correlated with RIB levels (*r* = 0.682, *p* = 0.003; Fig. 1d). The results indicate that a tight connection may exist between elevated RIB and depressive illness.

### RIB-fed mice exhibit depressive-like behaviors

**Fig. 2:**
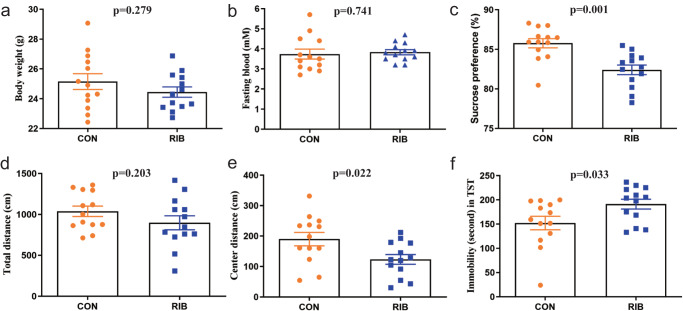
Behavioral experiments in the control (CON) and d-ribose (RIB) groups. **a**, **b** Both body weight (**a**) and fasting blood (**b**) were similar between the two groups. **c** Sucrose preference (%) was significantly lower in the RIB group. **d** Total distance was similar between the CON and RIB groups. **e** Center distance was significantly lower in the RIB group. **f** Compared to the CON group, the RIB group had a significantly higher immobility time. Data are the means ± standard error of mean, *n* = 13 per group. Student’s *t* test (data was normally distributed) or nonparametric Mann–Whitney *U* test (data was not normally distributed) was used to analyze the data.

After eight weeks of RIB treatment, there were no significant differences in the mouse body weight (*p* = 0.279; Fig. 2a) and fasting blood glucose (*p* = 0.741; Fig. 2b) between the CON and RIB groups. However, the result of sucrose preference was significantly lower in the RIB group than in the CON group (*p* = 0.001; Fig. 2c). In open field test, there was no difference in the total distance between the two groups (*p* = 0.203; Fig. 2d), whereas the central distance in the RIB group was significantly lower than that in the CON group (*p* = 0.022; Fig. 2e). Moreover, in tail suspension test, the RIB group had significantly higher immobility time than the CON group (*p* = 0.033; Fig. 2f). These group-level comparisons were conducted using Student’s *t* test. These results further confirm that RIB-fed induced depressive-like behaviors in mice.

### RIB impairs the intestinal epithelial barrier

**Fig. 3:**
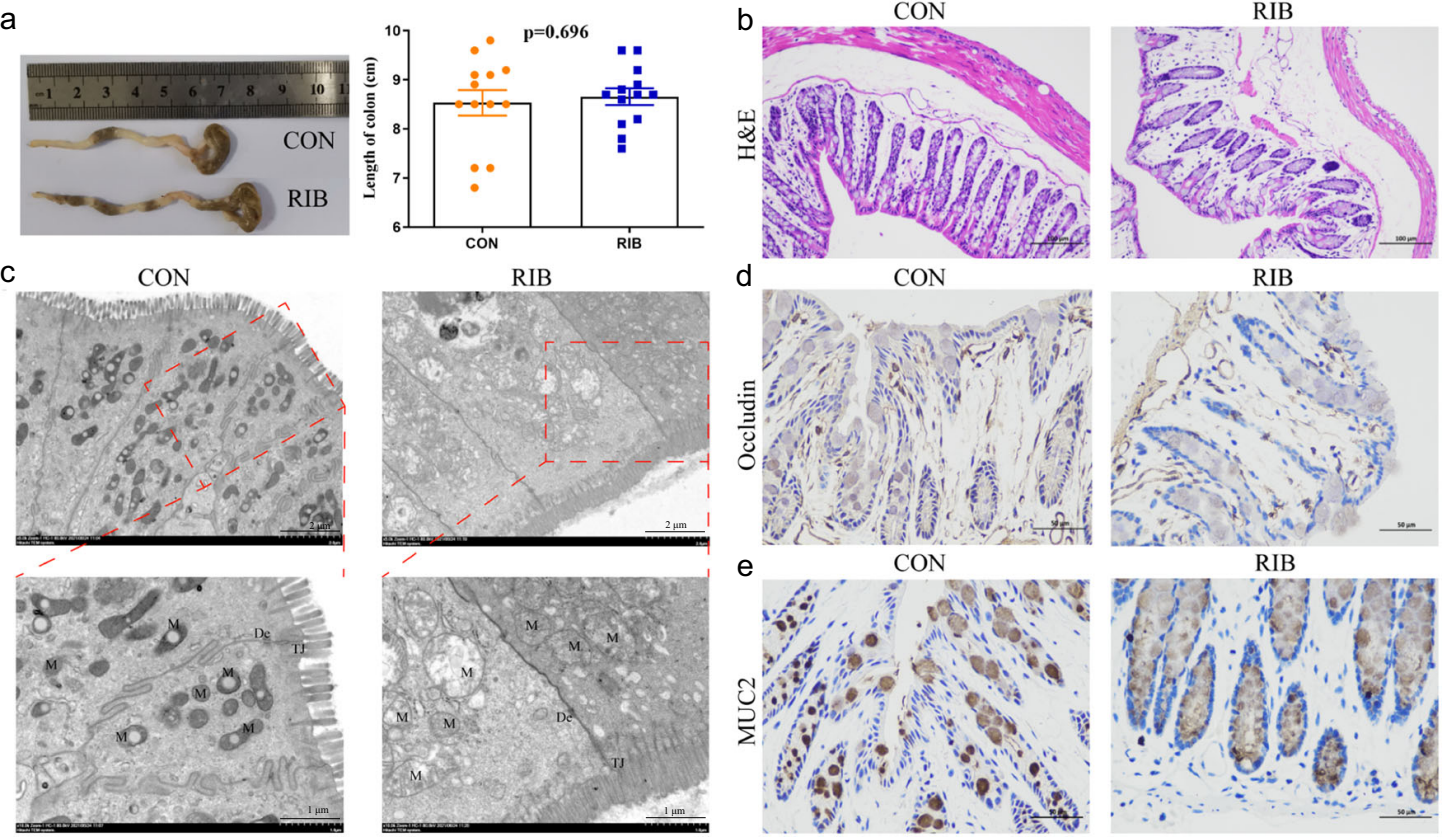
Colonic epithelial barrier histology analysis in control (CON) versus d-ribose (RIB)-fed mice. **a** Representative images of colons and quantification of colon length were statistically analyzed (Data are the means ± standard error of mean, *n* = 13 per group), and no significant difference in the length of the colon between the two groups (Student’s *t* test, *p* = 0.696). **b** Representative hematoxylin–eosin (H&E) staining image, scale bar = 100 µm. **c** Representative transmission electron micrographs of colon epithelial cells (scale bar = 2 µm). The second row’s enlarged images were from the first row in the area indicated with a dotted line box (scale bar = 1 µm). M mitochondria, TJ tight junction, De desmosome. **d**, **e** Immunohistochemistry analysis for Occludin (D) and mucin 2 (MUC2; E) in colon tissue (scale bar = 50 µm).

Homeostasis in the gut is important for brain function. We investigated whether RIB feeding perturbed colonic homeostasis, including colonic barrier and gut microbiota. There was no significant difference in the length of the colon between the two groups (Student’s *t* test, *p* = 0.696; Fig. 3a), whereas hematoxylin and eosin staining revealed the thickness of the muscularis mucosae significantly thinner, and loss of crypts and glands in the RIB-fed mice (Fig. 3b). Electron microscopy also showed severe mitochondrial swelling, injury of tight junction and gap junction domains, reduced numbers of the desmosome, and increased distance between adjacent epithelial cells in the colon of RIB-fed mice (Fig. 3c). Moreover, immunohistochemistry analysis indicated that the expression of Occludin (Fig. 3d) and mucin 2 (MUC2; Fig. 3e) were obviously decreased in RIB-fed mice.

### Gut microbiome alterations in RIB-fed mice

**Fig. 4:**
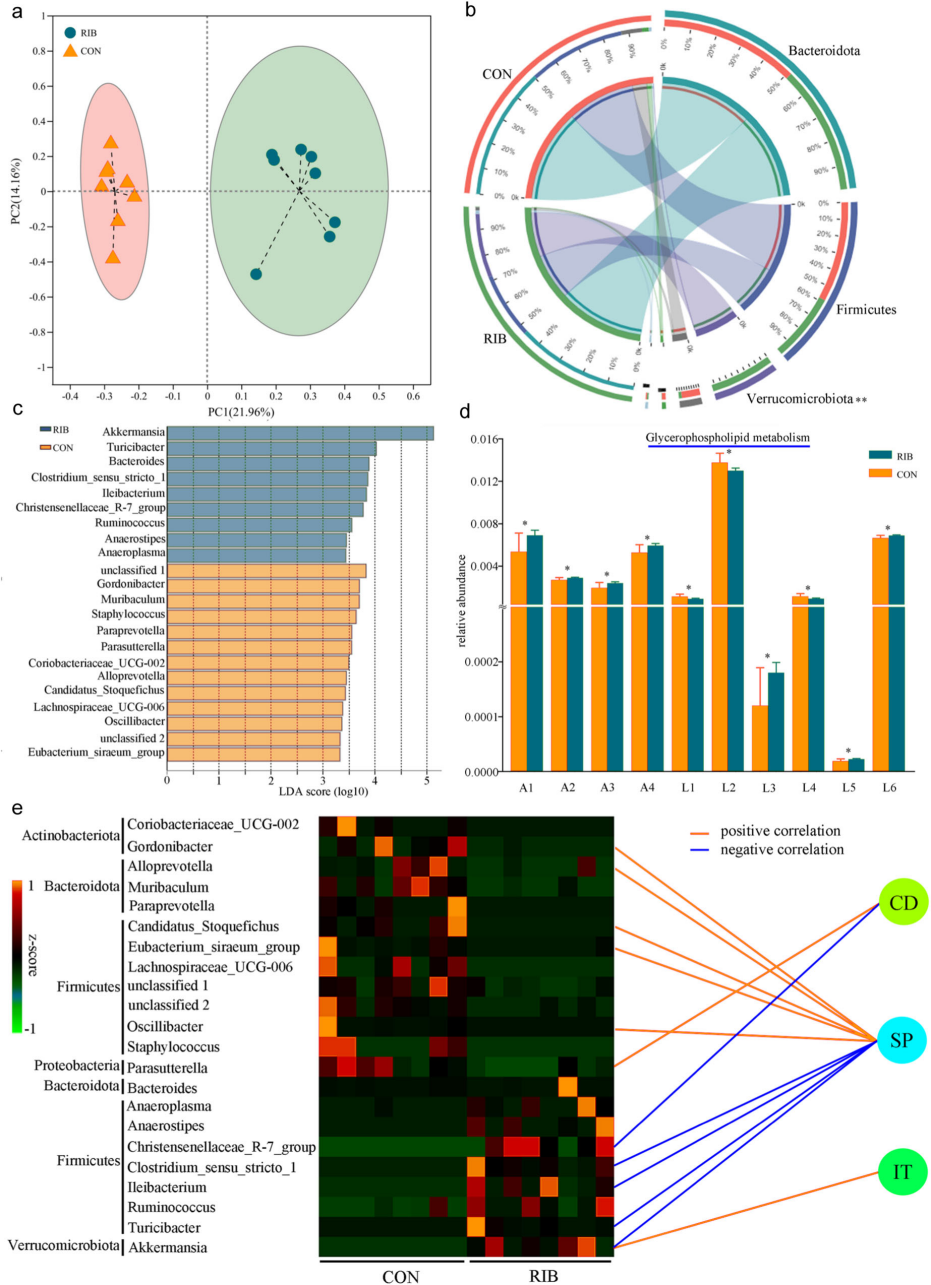
Gut microbiome differences in control (CON) versus d-ribose (RIB)-fed mice. **a** Principal coordinate analysis model showed that there were significantly differential gut microbiota compositions between CON and RIB-fed mice. **b** Firmicutes, Bacteroidetes, and Verrucomicrobiota were the three major bacterial phyla in both groups. **c** In total, 22 differential genera responsible for the discrimination between CON and RIB-fed mice were identified using linear discriminant analysis effective size. **d** These differential genera were significantly involved in four amino acid metabolism pathways and six lipid metabolism pathways (Student’s *t* test was used). The relative abundance of glycerophospholipid metabolism was highest among these pathways. A1: Phenylalanine, tyrosine, and tryptophan biosynthesis; A2: Lysine degradation; A3: Valine, leucine, and isoleucine biosynthesis; A4: Histidine metabolism; L1: Secondary bile acid biosynthesis; L2: Glycerophospholipid metabolism; L3: Arachidonic acid metabolism; L4: Primary bile acid biosynthesis; L5: Fatty acid elongation; L6: Fatty acid biosynthesis. **e** Correlations between differential genera and depressive-like behaviors (Spearman’s correlation analysis was used). CD center distance, IT immobility time, SP sucrose preference.

Next, gut microbiota diversity and composition in response to RIB were analyzed using 16S rRNA gene sequencing. There were no significant differences in alpha diversity between the two groups (Supplementary Fig. S1). The results of principal coordinate analysis showed that there were significant differences in beta diversity between the two groups (*p* = 0.001; Fig. 4a). As shown in Fig. 4b, Firmicutes and Bacteroidetes were the two major bacterial phyla in both groups and the relative abundance of Verrucomicrobiota was significantly higher in RIB-fed mice than in CON (*p* = 0.005). The relative abundances of each phylum were described in Supplementary Data 1.

Using linear discriminant analysis effective size, 22 differential genera responsible for the discrimination between CON and RIB-fed mice were identified (Fig. 4c and Supplementary Data 2). Most of the differential genera (*n* = 14, 63.63%) belonged to phylum Firmicutes. Kyoto Encyclopedia of Genes and Genomes (KEGG) pathway analysis (Fig. 4d) showed that these differential genera were significantly involved in four amino acid metabolism pathways (Phenylalanine, tyrosine, and tryptophan biosynthesis (up-regulate in RIB mice, *p* = 0.031); Lysine degradation (up-regulate in RIB mice, *p* = 0.030); Valine, leucine, and isoleucine biosynthesis (up-regulate in RIB mice, *p* = 0.037); Histidine metabolism (up-regulate in RIB mice, *p* = 0.029)) and six lipid metabolism pathways (Secondary bile acid biosynthesis (down-regulate in RIB mice, *p* = 0.031); Glycerophospholipid metabolism (down-regulate in RIB mice, *p* = 0.032); Arachidonic acid metabolism (up-regulate in RIB mice, *p* = 0.033); Primary bile acid biosynthesis (down-regulate in RIB mice, *p* = 0.031); Fatty acid elongation (up-regulate in RIB mice, *p* = 0.035); and Fatty acid biosynthesis (up-regulate in RIB mice, *p* = 0.030)).

To find out the differential genera significantly correlated with depressive-like behaviors, Spearman’s correlation analysis was conducted here. The results (Fig. 4e) showed that center distance was significantly correlated with Parasutterella (positive; *r* = 0.563, *p* = 0.032) and Christensenellaceae_R-7_group (negative; *r* = −0.513, *p* = 0.042), immobility time was significantly correlated with Akkermansia (negative; *r* = −0.519, *p* = 0.039), and sucrose preference was significantly correlated with nine differential genera (Candidatus_Stoquefichus (positive; *r* = 0.669, *p* = 0.005), Turicibacter (negative; *r* = −0.659, *p* = 0.006), Gordonibacter (positive; *r* = 0.627, *p* = 0.009), Eubacterium_siraeum_group (positive; *r* = 0.571, *p* = 0.021), Ileibacterium (negative; *r* = −0.551, *p* = 0.027), Clostridium_sensu_stricto_1 (negative; *r* = −0.541, *p* = 0.031), Alloprevotella (positive; *r* = 0.521, *p* = 0.039), Oscillibacter (positive; *r* = 0.518, *p* = 0.040), and Akkermansia (negative; *r* = −0.513, *p* = 0.042)).

### Differential microbial metabolites in RIB-fed mice

**Fig. 5:**
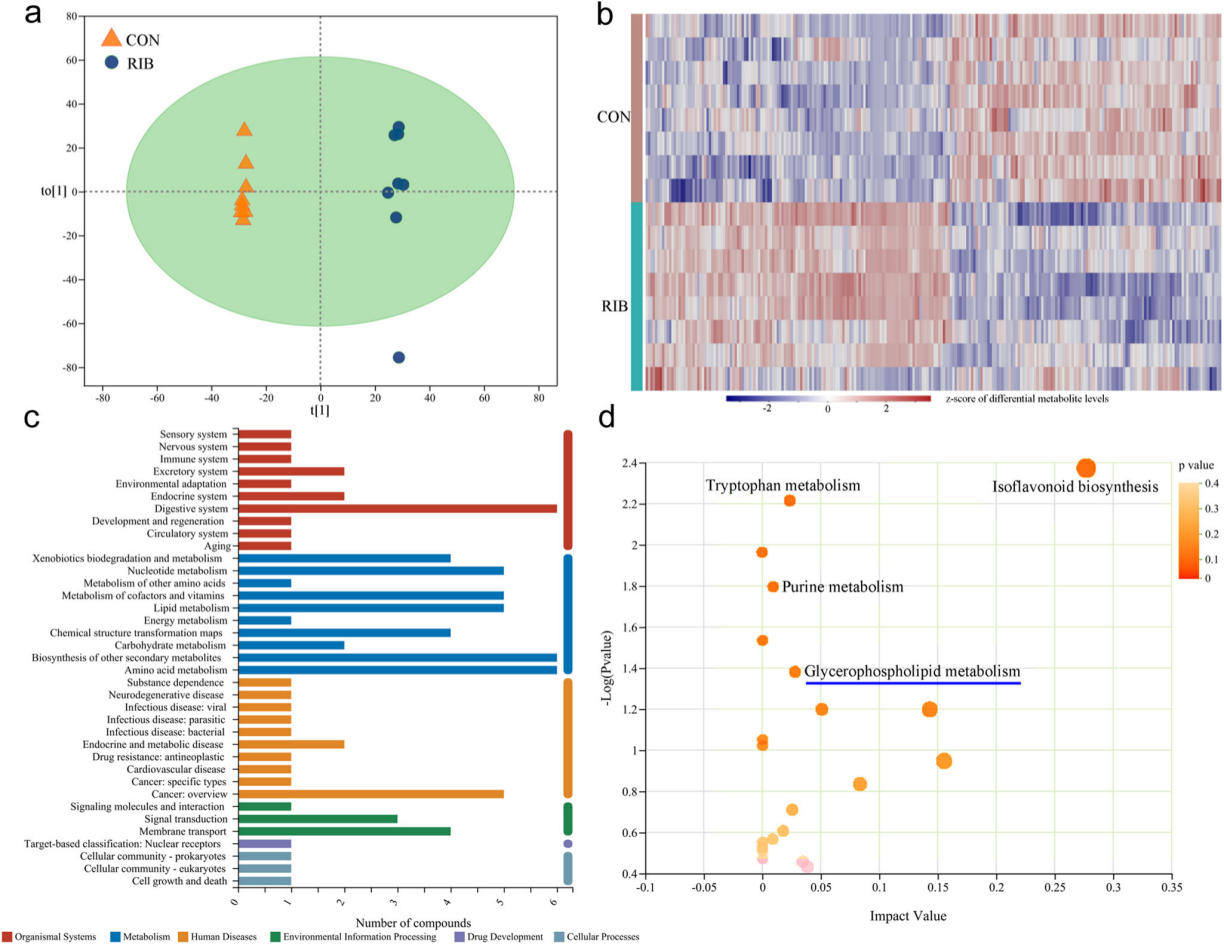
Divergent fecal metabolic phenotypes between the control (CON) and d-ribose (RIB) groups. **a** OPLS-DA model showed that the two groups had significantly different fecal metabolic phenotypes. **b** There were 246 differential fecal metabolites (VIP > 1.0 from corresponding OPLS-DA loading plots and *p* < 0.05 from Student’s *t* test) responsible for the discrimination between CON and RIB groups. **c** KEGG pathway classification showed that these metabolites were mainly annotated into the metabolism category. **d** Using online software MetaboAnalyst, four significantly dysregulated metabolic pathways in KEGG metabolism category classifications at level 3 were identified via hypergeometric test (each dot represents a KEGG path, the dot size represents the impact value, and the dot color represents the p-value, the more important the differential metabolites were in this pathway, the larger the dot).

In total, there were 1331 metabolites successfully annotated (Supplementary Data 3). The built orthogonal partial least-squares discriminant analysis (OPLS-DA) model using microbial metabolites in feces showed that the RIB-fed mice were separate from CON with no overlap, suggesting the divergent microbial metabolic phenotypes between the two groups (Fig. 5a). The results of 399-permutation testing demonstrated that this model was valid and not over-fitting (Supplementary Fig. S2). By analyzing the variable importance plot (VIP) from corresponding OPLS-DA loading plot and *p*-value from Student’s *t* test, we identified 246 differential microbial metabolites responsible for the discrimination between CON and RIB-fed mice (VIP > 1.0 and *p* < 0.05). Detailed information on these microbial metabolites was described in Supplementary Data 4. The heat map consisting of these differential microbial metabolites showed a consistent clustering pattern within the individual groups (Fig. 5b).

In addition, KEGG pathway classification showed that these differential microbial metabolites were mainly annotated into the metabolism category (Fig. 5c). Using online software MetaboAnalyst, four significantly affected metabolic pathways in KEGG metabolism category classifications at level 3 were identified via hypergeometric test (Fig. 5d): Tryptophan metabolism (*p* = 0.006), Isoflavonoid biosynthesis (*p* = 0.004), Purine metabolism (*p* = 0.016), and Glycerophospholipid metabolism (*p* = 0.041).

### Differential metabolites in the colon, blood, and hippocampus

**Fig. 6:**
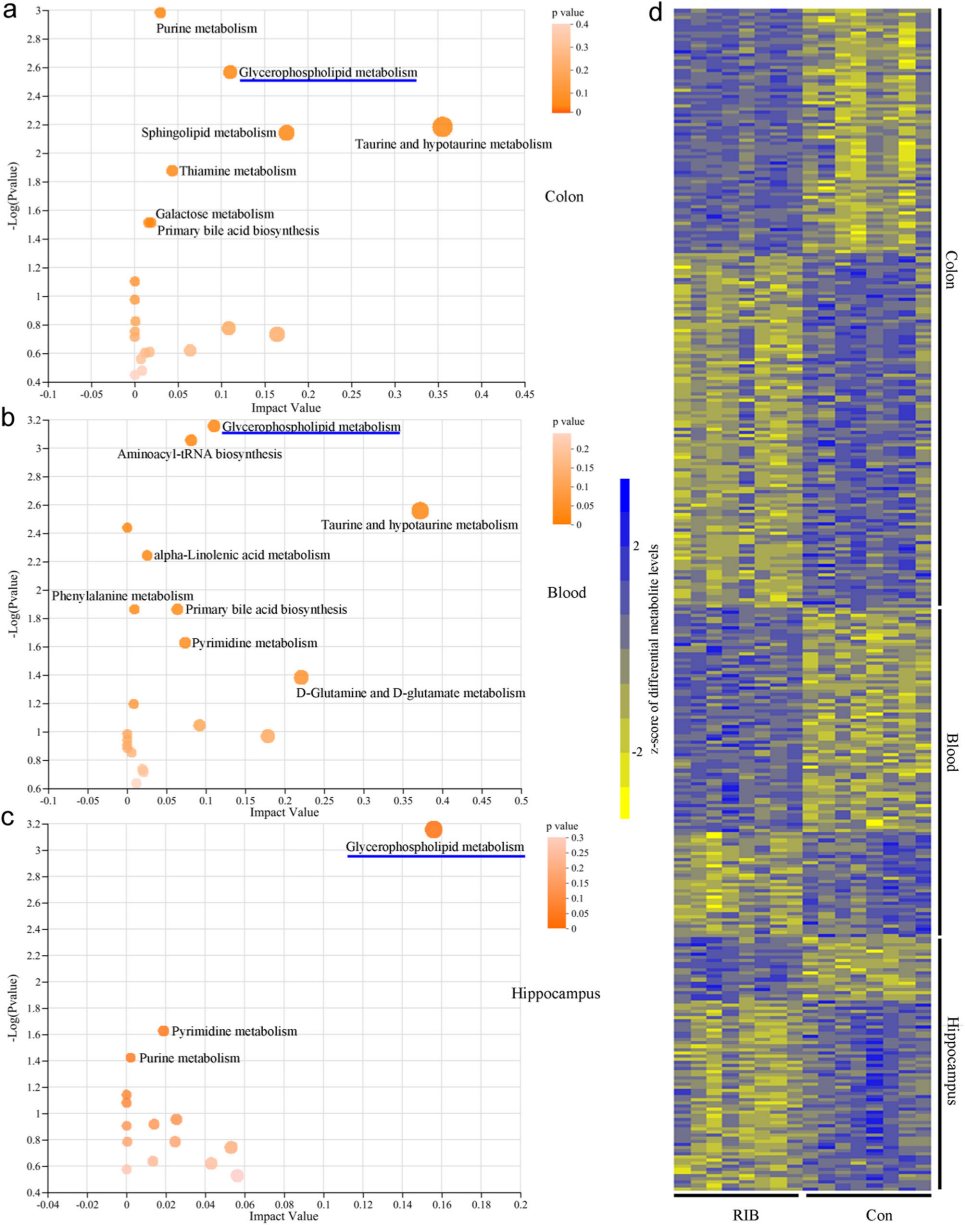
Metabonomic analysis of other biosamples in KEGG metabolism category classifications at level 3. **a** Seven significantly dysregulated metabolic pathways were found using hypergeometric tests in colon tissue. **b** Eight significantly dysregulated metabolic pathways were identified using hypergeometric tests in blood. **c** Three significantly dysregulated metabolic pathways were identified using hypergeometric tests in hippocampus. The online software MetaboAnalyst was used to conduct pathway analysis. Each dot represents a KEGG path, the dot size represents the impact value, and the dot color represents the *p*-value. The more important the differential metabolites were in this pathway, the larger the dot. **d** A heat map representation comprising all the differential metabolites from the colon, blood, and hippocampus showed a consistent clustering pattern within the individual groups.

In total, 1063 metabolites were successfully annotated in the colon (Supplementary Data 5). The built OPLS-DA model showed divergent metabolic phenotypes in the colon between the two groups (Supplementary Fig. S3a). There were 189 differential metabolites responsible for separating RIB-fed mice from CON were identified (VIP > 1.0 and *p* < 0.05; Supplementary Data 6). KEGG pathway classification showed that these differential microbial metabolites were mainly annotated into the metabolism category; and using the online software MetaboAnalyst, seven significantly affected metabolic pathways in KEGG metabolism category classifications at level 3 were identified via hypergeometric test (Fig. 6a): Galactose metabolism (*p* = 0.031), Glycerophospholipid metabolism (*p* = 0.003), Sphingolipid metabolism (*p* = 0.007), Primary bile acid biosynthesis (*p* = 0.031), Thiamine metabolism (*p* = 0.013), Taurine and hypotaurine metabolism (*p* = 0.007), and Purine metabolism (*p* = 0.001).

Similarly, in blood, 665 metabolites were successfully annotated (Supplementary Data 7), and 104 differential metabolites responsible for separating RIB-fed mice from CON were identified (VIP > 1.0 and *p* < 0.05; Supplementary Fig. S3b and Supplementary Data 8). Using online software MetaboAnalyst, eight significantly affected metabolic pathways in KEGG metabolism category classifications at level 3 were identified via hypergeometric test (Fig. 6b): Phenylalanine metabolism (*p* = 0.014), Glycerophospholipid metabolism (*p* = 0.001), Primary bile acid biosynthesis (*p* = 0.014), alpha-Linolenic acid metabolism (*p* = 0.006), Taurine and hypotaurine metabolism (*p* = 0.003), d-Glutamine and d-glutamate metabolism (*p* = 0.041), Pyrimidine metabolism (*p* = 0.024), and Aminoacyl-tRNA biosynthesis (*p* = 0.001).

In the hippocampus, 762 metabolites were successfully annotated (Supplementary Data 9), and 80 differential metabolites responsible for separating RIB-fed mice from CON were identified (VIP > 1.0 and *p* < 0.05; Supplementary Fig. S3c and Supplementary Data 10). Using online software MetaboAnalyst, three significantly affected metabolic pathways in KEGG metabolism category classifications at level 3 were identified via hypergeometric test (Fig. 6c): Glycerophospholipid metabolism (*p* = 0.001), Pyrimidine metabolism (*p* = 0.019), and Purine metabolism (*p* = 0.002). A heat map representation comprising all the differential metabolites from colon, blood, and hippocampus showed a consistent clustering pattern within the individual groups (Fig. 6d).

### Metabolomic correlations with behavioral phenotypes

**Fig. 7:**
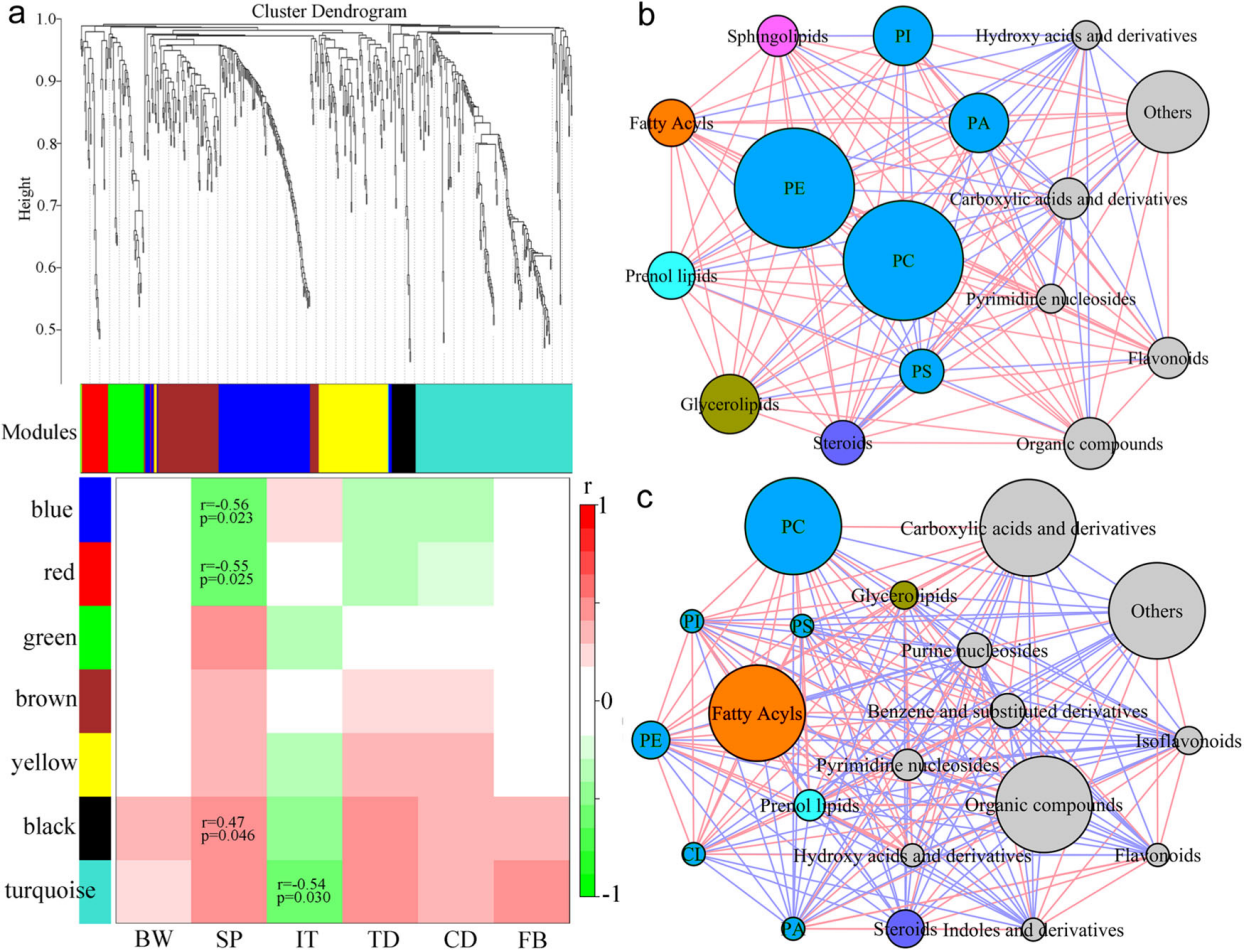
Metabolomic correlations with behavioral phenotypes using weighted correlation network analysis. **a** Spearman’s correlations between behavioral phenotypes and metabolomic modules. SP was significantly correlated with three metabolic modules (blue, red, and black), and IT was significantly correlated with the turquoise metabolic module. Red and green squares indicated positive and negative correlations, respectively. **b** The differential metabolites in the turquoise metabolic module were significantly correlated with IT mainly belonged to lipid metabolism, especially PE and PC. **c** The differential metabolites in three metabolic modules (blue, red, and black) were significantly correlated with SP mainly belonging to PC, fatty acyls, organic compounds, and carboxylic acids and derivatives. Differential metabolites belonging to lipid metabolism were marked using different colors except for the gray, and other differential metabolites were marked using gray circles. Circle size indicated the abundance of the metabolites belonging to this node. BW body weight, CD center distance, CL cardiolipin, FB fasting blood, IT immobility time, PA phosphatidic acid, PC phosphatidylcholine, PE phosphatidylethanolamine, PI phosphatidylinositol, PS phosphatidylserine, SP sucrose preference, TD total distance.

As the samples of the MGB axis were diverse, only a limited number of metabolites (*n* = 17) were shared by these detected samples (feces, colon, blood, and hippocampus). This phenomenon was also observed in our previous nonhuman primate model of depression^22^. As such, the different components of a given metabolic pathway might synergistically modulate the function of the MGB axis in different tissues. Thus, weighted correlation network analysis was used here to cluster the identified differential metabolites into the metabolic modules of the MGB axis. The results showed that there were seven different modules, in which four modules (blue, red, black, and turquoise) were significantly correlated with at least one type of depressive-like behavior (Fig. 7a).

Module-trait analysis showed that the differential metabolites in the turquoise metabolic module significantly correlated with immobility time were mainly involved in peripheral and central glycerophospholipid metabolism within the MGB axis (Fig. 7b); and the differential metabolites in the other three metabolic modules (blue, red, and black) significantly correlated with sucrose preference were mainly involved in peripheral and central glycerophospholipid and fatty acyls metabolism within the MGB axis (Fig. 7c). Details regarding the module and chemical class of each compound were shown in Supplementary Data 11.

### Correlations between differential genera and glycerophospholipids

**Fig. 8:**
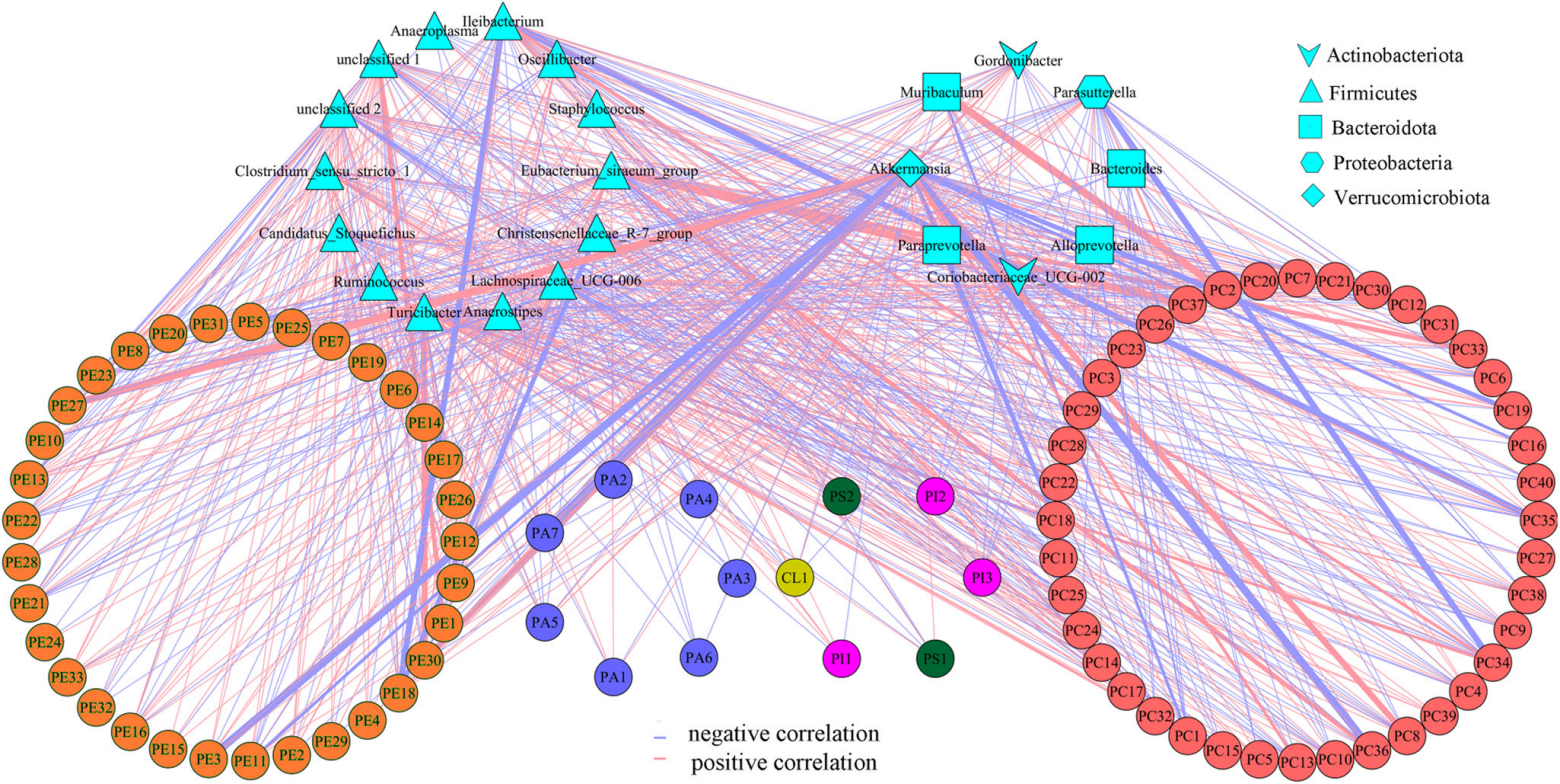
Correlations between differential genera and glycerophospholipids. The results of Spearman’s correlation analysis showed that the differential bacteria taxa mainly correlated with differential metabolites belonging to PE and PC. The numbers associated with the metabolite names were just codes, for example, we used PC39 to represent 1-heptadecanoyl-glycero-3-phosphate (detailed information about the codes representing metabolites is shown in Supplementary Data 12). PC phosphatidylcholine, PE phosphatidylethanolamine.

The abovementioned findings indicated that glycerophospholipid metabolism might play an important role in the crosstalk of gut microbiota and the brain. Thus, we further analyzed the potential correlations between differential genera and glycerophospholipids using Spearman’s correlation analysis. The results (Fig. 8) showed that there were significant correlations between differential genera and glycerophospholipids, especially metabolites belonging to phosphatidylcholine (PC) and phosphatidylethanolamine (PE).

**Fig. 9:**
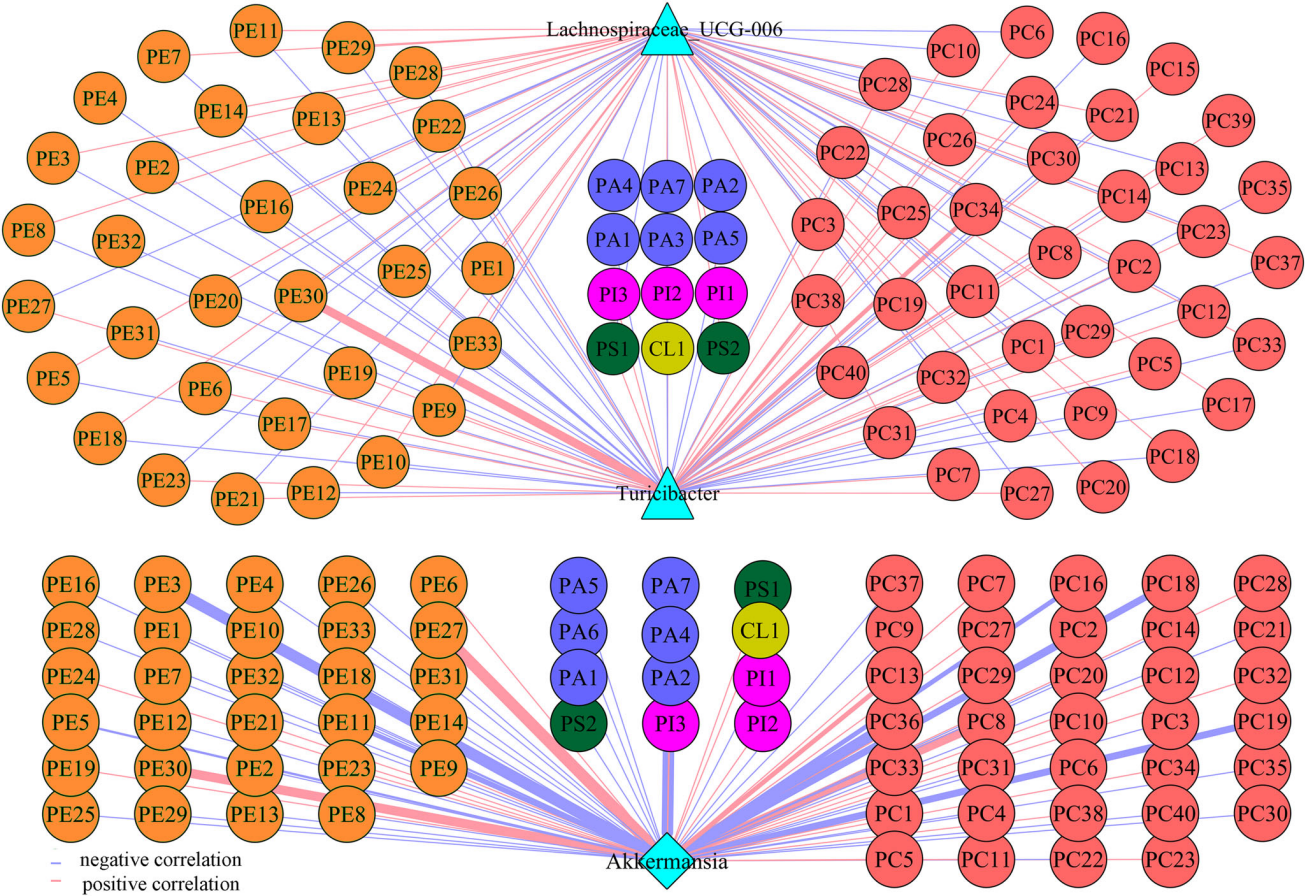
Correlations between differential genera and differential metabolites. Correlations between three differential genera (Lachnospiraceae_UCG-006, Turicibacter, and Akkermansia) and two types of glycerophospholipids (PC and PE) had greater contributions to the overall correlation between these differential genera and differential metabolites. The numbers associated with the metabolite names were just codes, for example, we used PC39 to represent 1-heptadecanoyl-glycero-3-phosphate (detailed information about the codes representing metabolites is shown in Supplementary Data 12). PC phosphatidylcholine, PE phosphatidylethanolamine. Spearman’s correlation analysis was used here.

Further analysis found that correlations between three differential genera (Lachnospiraceae_UCG-006, Turicibacter, and Akkermansia) and two types of glycerophospholipids (PC and PE) had greater contributions to the overall correlation between differential genera and glycerophospholipids (Fig. 9). These results indicated that glycerophospholipid metabolism, especially PC and PE, might be the important bridge of gut microbiota in affecting brain functions.

## Discussion

Dietary sugars, like fructose and glucose, are associated with psychosis-related higher brain dysfunctions^12,15^. Our previous study provided evidence that another simple sugar, RIB, could lead to depressive-like behaviors, and we demonstrated in mice that this was connected with altered hippocampus metabolic and transcriptome profiles^13^, but how the brain is affected by RIB remains poorly understood. In this study, we clarified that the RIB level was significantly increased in the depressed patients and depression model rats, and there was an obvious correlation between the change of RIB and the severity of depression disorder. However, these studies only involved untargeted metabolomics analysis, which provides relative metabolite abundance rather than absolute quantification^23^. The results further suggested that high levels of RIB were correlated with depression. We observed the RIB-fed mice were characterized by intestinal epithelial barrier impairment, alterations of microbial composition, function, and metabolic pathways of the MGB axis. Meanwhile, the altered microbial and metabolic modules linked the gut microbiome with dysregulation of peripheral and hippocampus glycerophospholipid metabolism in RIB-fed mice. To our knowledge, this is the first report of RIB influencing gut microbiota, and gut dysbiosis may be responsible for mediating the depressive-like behaviors seen in RIB-fed mice by regulating the MGB metabolism.

We found that the RIB-fed mice had considerably impaired intestinal barrier as compared to the CON. The gut barrier function was regulated by the gut microbiota^24,25^. Gut homeostasis also has a significant role in maintaining the host’s health. Previous clinical studies have found that depressed individuals’ gut microbiomes have changed significantly^26,27^. As a result, we deduced that the RIB might disrupt the gut microbiota, and that the gut dysbiosis would subsequently lead to depression via the MGB axis. Using 16 S rRNA gene sequencing, we discovered that the RIB-fed mice were characterized by 22 differential bacteria taxa on the Genera level, especially Akkermansia, Turicibacter, and Lachnospiraceae_UCG-006. Akkermansia belonged to the Verrucomicrobiota phylum. Khan S et al.^28^ observed that feeding dietary simple sugars like glucose and fructose would enhance the abundance of Akkermansia, which is consistent with our current findings. The mucus-degrading bacterium Akkermansia would regulate intestinal homeostasis and preserve the integrity of the gut barrier^29^. The increased Akkermansia might be the cause of intestinal barrier impairment in RIB-fed mice. Interestingly, recent research has shown that Akkermansia has beneficial roles in human health^30^; nevertheless, there is also strong evidence that Akkermansia promotes the etiology of colitis^31^. Similarly, Akkermansia was detected in much higher abundance in individuals with severe depressive symptoms, according to Zhang et al.^32^, while Ding et al.^33^ suggested that Akkermansia might ameliorate chronic stress-induced depressive-like behaviors. These contradictory findings might be the consequence of differences in participants, sequencing, and analytical approaches. Turicibacter and Lachnospiraceae_UCG-006 belonged to phylum Firmicutes. Furthermore, we found that 63.63% of the differential genera belonged to the phylum Firmicutes. This result is in line with our earlier research that 44.44% of differential genera in depressed individuals also belonged to the phylum Firmicutes^34^. At the phylum level, disturbances of Firmicutes have been identified as a possible hallmark of depression^19,35^. Accordingly, these results suggested that RIB would induce the gut microbiota disordered, and that the bacterial phylum Firmicutes disturbances might be a significant contributing factor to RIB-caused depressive-like behaviors.

According to previous research^36^, RIB levels in human urine were positively correlated with serum triglyceride levels, and Sprague-Dawley rats given RIB injection had considerably higher hepatic triglyceride levels, suggesting that RIB might regulate lipid metabolism. Besides, lipids are crucial for brain neuronal activity^37^, and the lipid composition of the brain has a significant impact on emotional behavior and subjective mood^38^. It has been shown that depressed individuals’ peripheral and central lipid metabolism is disturbed^39,40^. Here, we found that both altered microbial and metabolic modules involving glycerophospholipid metabolism were highly correlated with depressive-like behaviors in RIB-fed mice. Our previous studies revealed that the gut microbiota would significantly affect the glycerophospholipid metabolism in the mouse brain^41,42^. Meanwhile, the glycerophospholipid metabolism in the hippocampus was disturbed in the chronic unpredictable mild stress rat model of depression^42^. Thus, we concluded that in RIB-fed mice, peripheral and central glycerophospholipid metabolism was regulated by gut dysbiosis, which might be a contributing factor to the development of depressive-like behaviors.

In addition, we found that PC and PE had greater contributions to the overall correlation between differential genera and glycerophospholipids. Our previous study has also shown that they are remarkably increased in depressed patients and have favorable associations with depression severity^43^. Moreover, PC and PE are critical components of neuronal membranes. The phospholipase A2 can deacylate PC and PE into lysophosphatidylcholine and lysophosphatidylethanolamine, which are then converted into glycerophosphocholine and glycerophosphoethanolamine. Patients with depression have increased amounts of the neurotransmitter acetylcholine, which is produced by glycerophosphocholine^44^. Higher levels of glycerophosphoethanolamine have been found in the white matter of depressed individuals^45^. In light of this, our findings suggested that in RIB-fed mice, PC and PE might act as a link between gut dysbiosis and depressive-like behaviors.

Several limitations of this study are listed as follows: First, it was conducted in mice; however, human gut microbiota compositions do not exactly match those of rodents, clinical trials are required to further confirm the reported effects of RIB on human gut microbiota composition and function. Second, we only focused on one region of the brain associated with emotions (the hippocampus), whereas chronic stress caused lipidomic changes in a region-specific manner, and the disturbances of lipid metabolism in the prefrontal cortex were more obvious than those in the hippocampus^46^. As a result, future studies should take other brain regions to uncover more novel clues on the interactions between gut microbiota and RIB-caused depression. Third, our data do not completely rule out the possibility of other direct effects of RIB on the host, even though we thoroughly defined host peripheral and hippocampus metabolism implicated in RIB-induced gut dysbiosis. Future study on fecal microbiota transplantation is needed to clarify how the MGB axis links RIB to aberrant emotional-associated behavior. Fourth, due to the dose of ribose being a constant here, we could not use mediation analyses to assess whether ribose directly induced depression or indirectly induced depression through influencing the gut microbiome or gut-brain axis glycerophospholipid metabolism. Future studies should explore the role of RIB (directly or indirectly) in depression by designing gradient RIB dose experiments or modulating the important differential variables identified in this study (bacterial taxa or metabolites). Fifth, we investigated the underlying pathogenesis using an integrated metabolomics and 16 S rRNA gene sequence analysis approach. Considering that RIB induced depressive-like behavior, which correlated with intestinal barrier damage and gut microbiota imbalance, the issue of whether intervention or reversal of intestinal barrier damage and gut microbiota imbalance could circumvent the effects of RIB requires further investigation in future studies. Sixth, due to the limitations of technologies and funds, we did not conduct further experiments to validate the functional relationship of RIB with the glycerophospholipids metabolism pathway.

In conclusion, to our knowledge, this is the first study to reveal that oral RIB results in depressive-like behaviors, which may be partially explained by changes in microbial composition, function, and metabolism of the MGB axis. The disturbances of gut microbiota by RIB, especially three differential genera (Lachnospiraceae_UCG-006, Turicibacter, and Akkermansia), may contribute to the onset of depressive-like behaviors via regulating two types of glycerophospholipids (phosphatidylcholine and phosphatidylethanolamine) metabolism. This study highlights that simple sugars like RIB can have adverse effects on gut microbiota, MGB axis metabolism, and mental health.

## Methods

### Analysis of the change of RIB in depressed patients and depression model rats

Using our group’s previous metabonomics data on depressed patients^47^ and animal models^48^, an analysis of RIB levels was performed in the urine of patients with major depressive disorder (MDD; *n* = 126) and HC (*n* = 125), and in the hippocampus from chronic social defeat stress model rats (*n* = 9) and CON rats (*n* = 8). The relative abundance of RIB was defined as RIB level in metabonomics. The MDD patients were recruited from the Psychiatric Center of the First Affiliated Hospital of Chongqing Medical University, and the HC were recruited from the Medical Examination Center of the First Affiliated Hospital of Chongqing Medical University. All included individuals were ethnically homogenous Han Chinese. The Hamilton Depression Scale score of MDD patients has to be more than 17, and all of them were first-episode drug-naïve MDD patients. The human data used and study protocol have received ethical approval from the Medical Research Ethics Committee of Chongqing Medical University. All ethical regulations relevant to human research participants were followed, and a statement confirming that informed consent was obtained.

Following the protocol^49^, a typical depression phenotype caused by chronic mild stress was modeled in rats using a chronic social defeat stress paradigm. In brief, an “intruder” (Sprague-Dawley rat) was introduced into the home cage of the “resident” (aggressive male Long-Evans rat). The interaction continued until the intruder received a serious defeat, with a maximal interaction time of 5 min. Then, the intruder was transferred to a wire mesh protection cage (10 × 10 × 15 cm) within the resident’s cage that allowed intense visual, auditory, and olfactory contact with the resident while not direct body contact for 55 min. Meanwhile, the control rats were exposed to the empty home cage of an aggressive Long-Evans rat for 60 min.

Moreover, to explore the possible sex effects, RIB levels in male (*n* = 63) and female (*n* = 63) MDD patients were independently compared to those in their corresponding HC (male, *n* = 77; female, *n* = 48). The Spearman’s correlation analysis was used to investigate the possible correlation between RIB level and depressive-like behaviors in depression model rats, including the percentage of sucrose preference in the sucrose preference test and the immobility time in the tail suspension test.

### Experimental animal

C57BL/6 J male mice (6-8 weeks old) were purchased from Chongqing Medical University’s experimental animal center. Animals were housed in a specific pathogen-free vivarium under standard conditions. Mice were randomly divided into the CON group and RIB group (both *n* = 13). All RIB group mice were administered the RIB (3.6 g/kg/d RIB (Sigma-Aldrich, St. Louis, MO, USA) in drinking water) for eight weeks. The dosage and time were determined by previously published studies^13,50^. The statement of the European Food Safety Authority also reported that RIB was safe for the general population at intake levels up to 252 g/d for a human body weight of 70 kg^5^, indicating that the dose of 3.6 g/kg/d RIB for human body was acceptable. Thus, here we used 3.6 g/kg/d RIB. The mice in the CON group were given access to normal purified water for feeding. Since food is one of the primary factors influencing changes in the gut microbiome, all the mice were fed the same food (standard laboratory mouse diet) to rule out any possible impacts. To reduce any environmental effects, all mice were kept in the same room and habituated to the experimental setting for at least a week, and the drinking water added RIB was replaced once every two days. In each group, eight mice were randomly selected for the 16S rRNA gene sequence and untargeted metabolomics analysis. All animal experiments were carried out following the U.K. Animals (Scientific Procedures) Act, 1986, and we have complied with all relevant ethical regulations for animal use. This study protocol has received ethical approval from the Institutional Animal Care and Use Committee of Chongqing Medical University.

### Body weight and fasting blood glucose detection

At the endpoint of RIB treatment, the mice’s body weights were recorded, and the metric was gram. Moreover, after fasting overnight, the blood glucose of each mouse was measured from the caudal vein using a blood glucose meter (Roche, Basel, Switzerland), the metric used here was mmol/L.

### Behavioral experiments

Mice were subjected to a series of behavioral tests in a blinded manner by two experimenters^13,51^. The observers had no knowledge of the mice groupings prior to the experiment. All behavioral tests were performed daily from 9:00 am to 4:00 pm with minimal stress. The interval was 24 h between tests to avoid the effects of the tail suspension test.

#### Sucrose preference test

Mice were adapted to a 1% sucrose solution for 3 days before being presented with two bottles that contained water or 1% sucrose solution on testing day. For sucrose preference, mice were given water and 1% sucrose solution, and 12 h consumption was quantified. The consumption of sucrose preference was calculated as follows: sucrose intake/total fluid consumption^52^.

#### Open field test

Mice were placed in an open field apparatus (50 × 50 × 40 cm) for 5 min 30 s, with the first 30 s used for adaptation. The locomotor activity was analyzed using the Noldus automated tracking system.

#### Tail suspension test

The test was performed, with the murine caudal tip adhered on a suture and suspended 30 cm above the ground. Each test lasted for 5 min 30 s, with the first 30 s used for adaption. The immobility duration of each mouse was counted by the Noldus automated tracking system.

### Colonic histopathology

Mice were dissected at the end of the experiments, and the entire colon was removed to measure its length from the colon-cecal junction to the anal verge. Excised colon tissue was fixed in 4% paraformaldehyde and embedded in paraffin, similar to the earlier description^13^, the blocks were serially cut into 4-μm-thick sections and stained with hematoxylin and eosin. Histological images were captured using an E100 microscope (Nikon, Tokyo, Japan) to explore the examine colon tunica mucosa, tunica submucosa, and tunica muscularis thickness.

### Transmission electron microscopy analysis

Excised colon tissues were dissected into small pieces (1 × 1 × 1 mm), and fixed in 1% osmium tetroxide for 2 h. Following the dehydration steps, the tissues were embedded in Epon 812 resin (Electron Microscopy Sciences; Hartfield, PA, US), sliced, and stained with 2% uranyl acetate and lead citrate. Finally, the slices were observed with an electron microscope (HT7700, HITACHI, Tokyo, Japan), and the images were captured on a Morada camera (Münster, Germany).

### Histology

Briefly, 4% paraformaldehyde-fixed and paraffin-embedded colon tissues were sectioned into 4μm sections. The intestinal epithelial barrier markers Occludin (1:200, Abcam, ab216327) and colonic MUC2 (1:2000, Abcam, ab272692) were then detected by immunohistochemistry^53^. All images were captured using a Nikon DS-U3 microscope (Nikon, Tokyo, Japan).

### 16S rRNA gene sequence analysis

The EZNA-soil DNA kit (Omega Bio‐Tek, USA) was used to extract microbial DNA from frozen fecal samples in accordance with the manufacturer’s protocols and our previous studies^19,54^. DNA concentration and purity were assessed using the NanoDrop 2000 UV–vis spectrophotometer, and the quality of the DNA was verified using 1% agarose gel electrophoresis. The 16S rRNA gene of bacteria was amplified by Polymerase Chain Reaction using primers 338 F (5′‐ACTCCTACGGGAGGCAGCAG‐3′) and 806 R (5′‐GGACTACHVGGGTWTCTAAT‐3′) to target the V3–V4 hypervariable regions. AxyPrep DNA Gel Extraction Kit (Axygen Biosciences, Union City, CA, USA) was used to extract and purify amplicons from 2% agarose gels. Purified amplicons were quantified using QuantiFluor‐ST (Promega, USA) and paired‐end sequenced (2 × 250) on an Illumina MiSeq platform using standard protocols in Shanghai Majorbio Bio‐pharm Technology Co., Ltd. Trimmomatic was used to quality-filter raw fastq files, and short reads were quickly joined by adjusting their length. Using UPARSE (https://drive5.com/uparse/), the remaining high‐quality sequences were grouped into OTUs at 97% similarity. The ribosomal database project classifier algorithm (https://rdp.cme.msu.edu/) was used to examine the taxonomy of each 16S rRNA gene sequence. Alpha diversity was assessed to estimate the microbial communities’ diversity, including four parameters (Shannon, Simpson, Shao, and Phylogenetic diversity). Beta diversity analysis was performed to evaluate the difference in bacterial communities between CON and RIB using principal coordinate analysis plots. The linear discriminant analysis effective size is conducted to identify the key bacterial taxa responsible for discrimination between two groups.

### Untargeted metabolomics analysis

Similar to our previous studies^55,56^, peripheral and hippocampus tissue samples were prepared by homogenization, dissociation, and centrifugation. Serum samples were collected and centrifuged twice. L-2-chlorophenylalanine dissolved in methanol (0.02 mg/mL) was served as an internal standard. The untargeted liquid chromatography–mass spectrometry metabolomics analysis was conducted on a Thermo UHPLC-Q Exactive HF-X system equipped with an ACQUITY UPLC T3 column (100 mm × 2.1 mm i.d., 1.8 μm; Waters, USA). For the electrospray ionization positive mode, the mobile phases consisted of 0.1% formic acid in water (solvent A) and 0.1% formic acid in acetonitrile (solvent B). For the electrospray ionization negative mode, the mobile phases were water with 6.5 mM ammonium bicarbonate (solvent C) and 6.5 mM ammonium bicarbonate 95% methanol solution (solvent D). The flow rate was 0.40 mL/min and the column temperature was 40 °C. The injection volume was 2 μL. The mass spectrometric data was collected using the Thermo UHPLC-Q Exactive HF-X Mass Spectrometer equipped with an electrospray ionization source operating in positive mode and negative mode. The optimal conditions were set as follows: source temperature, 425 °C; sheath gas flow rate, 50 arb; Aux gas flow rate, 13 arb; ion-spray voltage floating, -3500 V (-) and 3500 V (+); Normalized collision energy, 20-40-60 V rolling for mass spectrometry/mass spectrometry.

The raw data generated by liquid chromatography–mass spectrometry were processed by baseline filtering, peak identification, integration, retention time correction, peak alignment, and normalization using Progenesis QI (Waters Corporation). The quality control samples were used to validate the stability of the metabolomic analysis. Metabolites were identified by Human Metabolome Database (http://www.hmdb.ca), Metlin (https://metlin.scripps.edu), and self-built databases. The peak intensity was deemed as the expression level of metabolite^57^. Unsupervised principal component analysis and OPLS-DA were used to show overall metabolic differences and variation between groups. Metabolite sets enrichment analysis was further performed based on the differential expressed metabolites using MetaboAnalyst 5.0 (http://www.metaboanalyst.ca)^58^. The differential metabolites were also inspected into the KEGG database (http://www.genome.jp/kegg/), and then KEGG was used to annotate the functions in which these differential metabolites were closely involved.

### Statistical and reproducibility

Statistical analyses were performed using SPSS 20 (Chicago, IL, US) and R studio (version 3.6.0, 2021). Data are presented as mean ± Standard Error of mean. *p* < 0.05 was considered statistically significant. The Student’s *t*-test, nonparametric Mann-Whitney *U* test, Chi-Square test, or Spearman’s correlation analysis was used when appropriate. Linear discriminant analysis effective size analysis was performed to identify the differential genera (linear discrimination analysis >2.0 and *p* < 0.05) responsible for the discrimination between groups. Phylogenetic Investigation of Communities by Reconstruction of Unobserved States was further used to predict the functions of these differential genera. Metabolites with VIP > 1.0 and *p* < 0.05 were viewed as differential metabolites by analyzing the OPLS-DA loading plot. To find out the key depressive-like behaviors-related metabolic modules of the MGB axis, the weighted correlation network analysis was used^59,60^. All data collection and analyses were performed blind to the conditions of the experiments. Sample sizes are defined in the corresponding figure legend. Especially, for the 16 S rRNA gene sequencing and metabolomics analysis, 16 mice were used (*n* = 8 in CON group, *n* = 8 in RIB group). For the detection of the hematoxylin and eosin, immunohistochemistry, and electron microscopy analysis, three biological independent animals were used.

### Supplementary information

Supplementary Information

Description of Additional Supplementary Files

Supplementary Data 1-2

Supplementary Data 3-12

Supplementary Data 13

## Data Availability

Raw data for 16S rRNA sequencing have been deposited in NCBI, under SRA accession number PRJNA1052466. Raw metabolite data are available in the Metabolights Database (MTBLS9185). Source data for Figs. 1, 2, and 3a are provided in Supplementary Data 13. All other data are available from the corresponding author upon reasonable request.
