# Supplementary material for: Oral d-ribose causes depressive-like behavior by altering glycerophospholipid metabolism via the gut-brain axis
Source: Commun Biol. 2024 Jan 9;7:69. doi: 10.1038/s42003-023-05759-1 (PMC10776610; doi:10.1038/s42003-023-05759-1)
Supplement: Supplementary file 1 — Supplementary Information [file 42003_2023_5759_MOESM1_ESM.pdf]

Oral D-ribose causes depressive-like behavior by altering glycerophospholipid metabolism via the gut-brain axis

Supplemental Information

Supplementary figures and tables

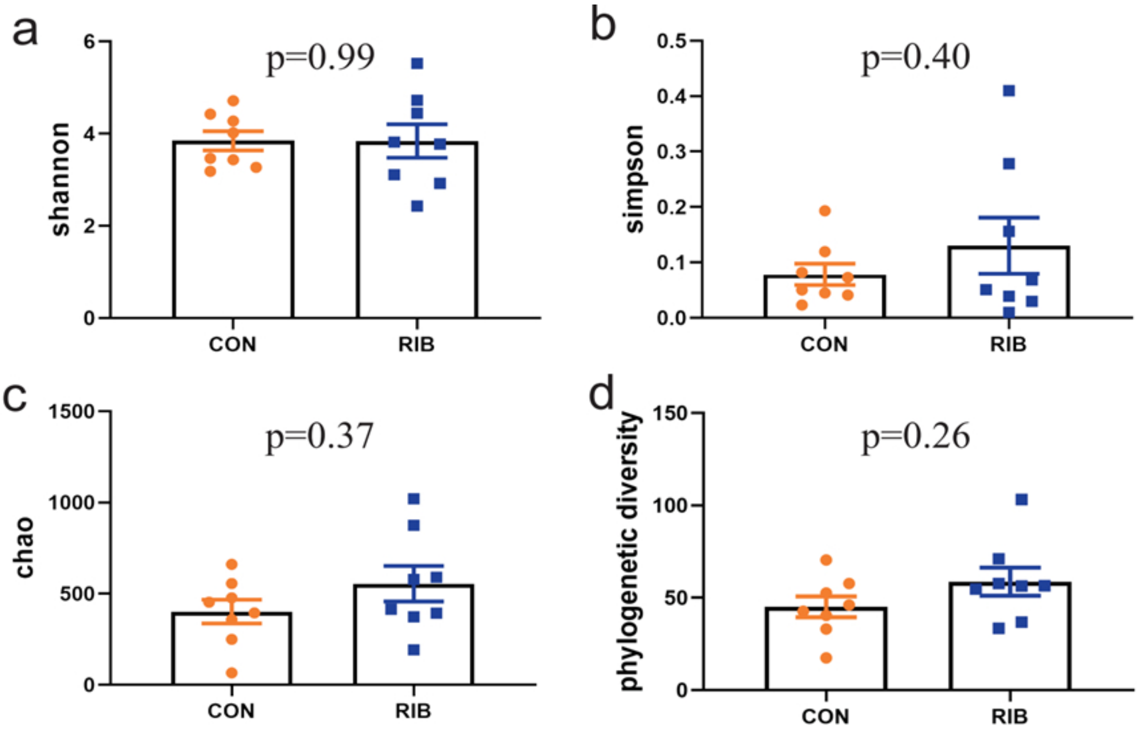

Fig. S1. Alpha diversity (shannon (a); simpson (b); chao (c); phylogenetic diversity (d)) a nalysis between the control (CON) and D-ribose (RIB) groups. Data are the means ± SEM, n = 8 per group.

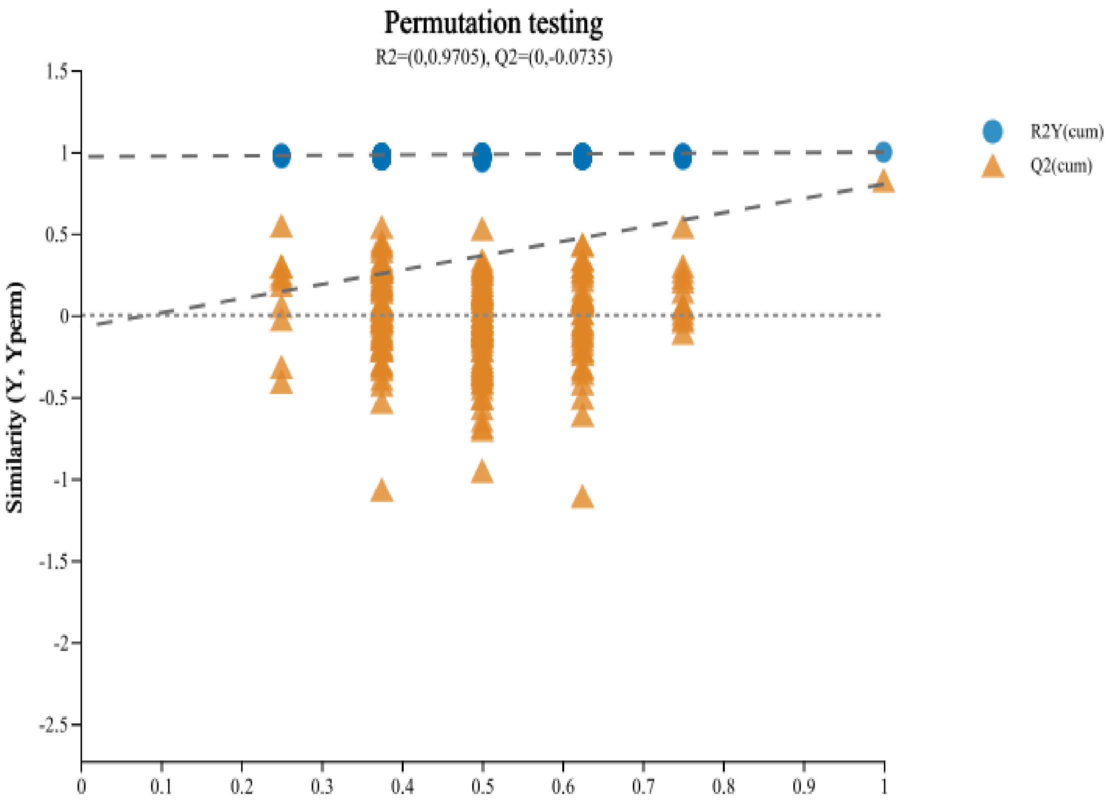

Fig. S2. 399-permutation testing. The plot above strongly indicated that the built OPLS-DA model was valid because the orange regression line of the Q2-points intersected the vertical axis (on the left) below zero (Q2=(0, -0.0735)).

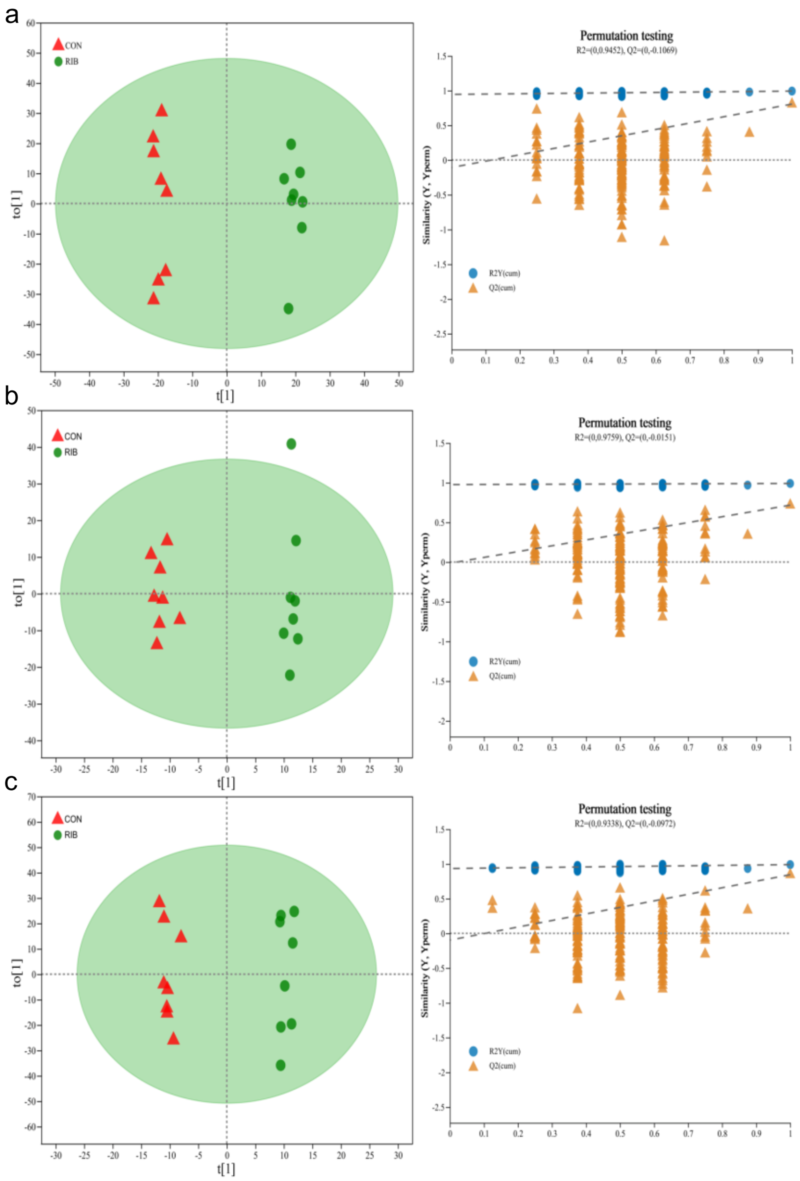

Fig. S3. OPLS-DA model and 399-permutation testing. (a) OPLS-DA model built using metabolite in the colon, the plot of permutation testing strongly indicated that the built OPLS-DA model was valid because the orange regression line of the Q2-points intersected the vertical axis (on the left) below zero (Q2=(0, -0.1069)). **(b)** OPLS-DA model built using metabolite in blood, the plot of permutation testing strongly indicated that the built OPLS-DA model was valid because the orange regression line of the Q2-points intersected the vertical axis (on the left) below zero (Q2=(0, -0.0151)). **(c)** OPLS-DA model built using metabolite in the hippocampus, the plot of permutation testing strongly indicated that the built OPLS-DA model was valid because the orange regression line of the Q2-points intersected the vertical axis (on the left) below zero (Q2=(0, -0.0972)).
