# Supplementary material for: Oral d-ribose causes depressive-like behavior by altering glycerophospholipid metabolism via the gut-brain axis
Source: Commun Biol. 2024 Jan 9;7:69. doi: 10.1038/s42003-023-05759-1 (PMC10776610; doi:10.1038/s42003-023-05759-1)
Supplement: Supplementary file 2 — Description of Additional Supplementary Files [file 42003_2023_5759_MOESM2_ESM.docx]

**File name: Supplementary Data 1**

**Description:** The relative abundances of each phylum between the control (CON) and D-ribose (RIB) groups.

**File name: Supplementary Data 2**

**Description:** Relative abundances of the differential genera between the control (CON) and D-ribose (RIB) groups.

**File name: Supplementary Data 3**

**Description:** All annotated metabolites in mice feces**.**

**File name: Supplementary Data 4**

**Description:** Relative abundances of the differential microbial metabolites between the control (CON) and D-ribose (RIB) groups**.**

**File name: Supplementary Data 5**

**Description:** All annotated metabolites in mice colon**.**

**File name: Supplementary Data 6**

**Description:** Relative abundances of the differential metabolites in the colon between the control (CON) and D-ribose (RIB) groups**.**

**File name: Supplementary Data 7**

**Description:** All annotated metabolites in mice serum.

**File name: Supplementary Data 8**

**Description:** Relative abundances of the differential metabolites in serum between the control (CON) and D-ribose (RIB) groups.

**File name: Supplementary Data 9**

**Description:** All annotated metabolites in mice hippocampus.

**File name: Supplementary Data 10**

**Description:** Relative abundances of the differential metabolites in the hippocampus between the control (CON) and D-ribose (RIB) groups.

**File name: Supplementary Data 11**

**Description:** Details information about the module and chemical class of each compound in weighted correlation network analysis.

**File name: Supplementary Data 12**

**Description:** Detail information about the codes that represent metabolites.

**File name: Supplementary Data 13**

**Description:** Source data for Fig. 1, Fig. 2, and Fig. 3a.
