# Supplementary material for: Oral d-ribose causes depressive-like behavior by altering glycerophospholipid metabolism via the gut-brain axis
Source: Commun Biol. 2024 Jan 9;7:69. doi: 10.1038/s42003-023-05759-1 (PMC10776610; doi:10.1038/s42003-023-05759-1)
Supplement: Supplementary file 3 — Supplementary Data 1-2 [file 42003_2023_5759_MOESM3_ESM.docx]

**Supplementary Data 1. The relative abundances of each phylum between the control (CON) and D-ribose (RIB) groups.**

| Phylum | CON | RIB | p value |
| --- | --- | --- | --- |
| Firmicutes | 1137.875 (651.624) | 773 (408.94) | 0.201 |
| Actinobacteriota | 242.25 (325.94) | 38.625 (17.229) | 0.099 |
| Cyanobacteria | 2.375 (3.962) | 1 (1.069) | 0.359 |
| Bacteroidota | 1557.125 (709.153) | 1444.625 (424.729) | 0.706 |
| Deferribacterota | 1 (1.773) | 0.125 (0.354) | 0.193 |
| Proteobacteria | 80 (202.912) | 5 (5.345) | 0.314 |
| Verrucomicrobiota | 0.5 (1.414) | 748.5 (733.204) | 0.005 |
| Desulfobacterota | 4.25 (6.228) | 3.875 (4.673) | 0.894 |
| unclassified phylum | 9.625 (5.975) | 16.625 (16.221) | 0.271 |
| Campilobacterota | 3.125 (3.563) | 11.75 (27.249) | 0.39 |
| Patescibacteria | 11.875 (14.076) | 6.875 (6.999) | 0.384 |

**Supplementary Data 2.** **Relative abundances of the differential genera between the control (CON) and D-ribose (RIB) groups.**

| Genera | CON | RIB | LDA value | p value |
| --- | --- | --- | --- | --- |
| Akkermansia | 0.5 (1.414) | 748.25 (732.878) | 5.129 | 0.0005 |
| Alloprevotella | 17.875 (21.781) | 3.75 (9.099) | 3.451 | 0.0283 |
| Anaeroplasma | 0.125 (0.354) | 3.5 (5.398) | 3.44 | 0.0256 |
| Anaerostipes | 0 (0) | 2.75 (4.234) | 3.452 | 0.0273 |
| Bacteroides | 15.25 (8.311) | 88.75 (240.544) | 3.885 | 0.0356 |
| Candidatus_Stoquefichus | 8.75 (12.635) | 0 (0) | 3.431 | 0.0107 |
| Christensenellaceae_R-7_group | 0 (0) | 2 (1.773) | 3.775 | 0.0038 |
| Clostridium_sensu_stricto_1 | 0.125 (0.354) | 38.125 (49.787) | 3.864 | 0.002 |
| Coriobacteriaceae_UCG-002 | 2.5 (4.408) | 0 (0) | 3.499 | 0.0273 |
| Eubacterium_siraeum_group | 6 (12) | 0.25 (0.707) | 3.328 | 0.0279 |
| Gordonibacter | 5.125 (6.958) | 0.125 (0.354) | 3.7 | 0.0256 |
| Ileibacterium | 0 (0) | 40.875 (46.094) | 3.838 | 0.0012 |
| Lachnospiraceae_UCG-006 | 3 (3.546) | 0 (0) | 3.381 | 0.0107 |
| Muribaculum | 19.25 (16.534) | 1.125 (2.475) | 3.699 | 0.0044 |
| Oscillibacter | 7 (17.809) | 0 (0) | 3.365 | 0.0273 |
| Paraprevotella | 2 (3.742) | 0 (0) | 3.555 | 0.0273 |
| Parasutterella | 6.625 (5.012) | 1 (1.414) | 3.553 | 0.0043 |
| Ruminococcus | 3.75 (8.697) | 20 (19.792) | 3.555 | 0.0153 |
| Staphylococcus | 1.875 (2.232) | 0 (0) | 3.639 | 0.0273 |
| Turicibacter | 0 (0) | 50.625 (106.811) | 4.026 | 0.0003 |
| unclassified 1 | 46.125 (43.096) | 10 (11.747) | 3.83 | 0.0156 |
| unclassified 2 | 7.125 (8.079) | 0.5 (0.926) | 3.329 | 0.0031 |
